# An Insight Into Systemic Immune Response in *Leishmania donovani* Mediated Atypical Cutaneous Leishmaniasis in the New Endemic State of Himachal Pradesh, India

**DOI:** 10.3389/fimmu.2021.765684

**Published:** 2022-01-04

**Authors:** Lovlesh Thakur, Priyanka Madaan, Aklank Jain, Vinay Shankar, Ajeet Negi, Shashi Bhushan Chauhan, Shyam Sundar, Om Prakash Singh, Manju Jain

**Affiliations:** ^1^ Department of Zoology, Central University of Punjab, Bathinda, India; ^2^ Department of Biochemistry, Central University of Punjab, Bathinda, India; ^3^ Department of Dermatology, Maharishi Markandeshwar Medical College and Hospital, Kumarhatti, Solan, India; ^4^ Department of Dermatology, Indira Gandhi Medical College, Shimla, India; ^5^ Department of Medicine, Institute of Medical Sciences, Banaras Hindu University, Varanasi, India; ^6^ Department of Biochemistry, Institute of Science, Banaras Hindu University, Varanasi, India

**Keywords:** atypical cutaneous leishmaniasis, *Leishmania donovani*, host immunity, Himachal Pradesh, India, cytokine profile, IgG isotyping

## Abstract

Leishmaniasis continues to afflict known and newer endemic sites despite global efforts towards its control and elimination. In this regard, the emergence of newer endemic sites with unusual disease formats is recognized wherein *Leishmania donovani* complex classically known to cause visceral disease is demonstrated to cause cutaneous manifestation. In this context, atypical cutaneous leishmaniasis (CL) cases caused by *L. donovani* genetic variants from the newer endemic state of Himachal Pradesh (HP) in India are beginning to be understood in terms of parasite determinants. The atypical CL manifestation further needs to be explored to define host immune correlates with a possible role in driving the unusual disease progression. In the given study, we performed comprehensive systemic-immune profiling of the atypical CL patients from the study area in HP, India, in comparison with the classical visceral leishmaniasis (VL) patients from the northeast region of India. The systemic immune response was studied using ELISA-based assessment of Th1, Th2, Th17, Treg, and Th22 specific plasma cytokine expression pattern and parasite-specific total serum IgG/IgG subclasses. The specified immune correlates are known to exhibit heterogeneous association with the different infecting parasite species, infection load, and co-lateral host immunopathology in classical CL and VL. In the atypical CL patient group, altered expression of IL-10 emerged as the key finding that could potentially fine-tune the Th1/Th17/Th22 effector cytokine axis towards a localized cutaneous manifestation. A reduced expression of IL-10 along with a high IFN-γ/IL-10 ratio as a readout of effective parasite killing defined atypical cutaneous outcome. In contrast, high circulatory IL-10 levels and a depressed IFN-γ/IL-10 ratio were seen in classical VL patients in line with an ineffective parasite-killing cytokine response. Overall, the study highlights new knowledge on host immune correlates in terms of cytokine expression pattern and IgG subclasses that underline atypical disease manifestation such that *L. donovani*, a generally visceralizing parasite species cause skin localized cutaneous lesions.

## Introduction

Leishmaniasis is an ongoing global public health problem despite efforts towards its control and elimination over the last decade. It is a disease complex with clinical manifestations ranging from systemic visceral leishmaniasis (VL) caused by *Leishmania donovani* complex, cutaneous leishmaniasis (CL) with skin restricted lesions caused by *Leishmania tropica* complex, and mucocutaneous leishmaniasis (MCL) involving mucocutaneous membrane caused by *Leishmania braziliensis* complex (1). The varied disease outcomes are determined by the infecting parasite species/subspecies and the host immune response. In the Indian subcontinent, classical systemic infection caused by anthroponotic *L. donovani* is the prominent disease form accounting for more than half the world’s disease burden in known and newer endemic regions in India, Nepal, Bangladesh, Bhutan, and Sri Lanka (2–5). CL cases are lesser in number with *L. tropica*/*L. major* as the causative agent in India, Nepal, and Bangladesh (6–8). More recently, the disease is emerging as an unusual cutaneous manifestation caused by *L. donovani* in the newer endemic sites in Sri Lanka, Nepal, and India (9–11). The emergence of such atypical CL poses a newer challenge to the ongoing disease-control program and thus needs to be studied to understand the atypical disease etiology. So far, there are few reports on genetic analysis of parasite isolates from atypical CL cases endemic in Sri Lanka, Nepal, and the states of Kerala and Himachal Pradesh (HP) in India, identified as *L. donovani* genetic variants distinct from the classical VL *L. donovani* isolates (9–12).

In regions of the emergence of dermotropic *L. donovani*, it is imperative to understand the immune response elicited by the parasite variants to further understand the unusual disease outcome. Immunology of VL and CL diseases caused by visceralizing versus dermotropic parasite species is well studied in a classical setting. *Leishmania* species-specific antigens prime and shape up the T-cell response, crucial in determining the course of the disease progression. In experimental models, a polarized Th1/Th2 helper T-cell response is seen with a Th1 skewed protective response in C57BL/6 resistant mice and a Th2-dominating disease progressive response in the susceptible BALB/c mice, while in humans, a mixed Th1/Th2 response is demonstrated (13, 14). Interestingly, the disease outcome is presently understood as a more complex phenomenon such that discrete parasite-specific antigens induce differential T-cell subset-specific inflammatory, anti-inflammatory, and/or regulatory cytokines that together fine-tune the disease outcome in terms of differential parasite load, dissemination, and persistence leading to a gradation of typical VL and CL manifestations (14–22).

Immune response in classical human VL cases is marked by elevated levels of multiple pro-inflammatory cytokines IFN-γ, TNF-α, IL-12, and IL-17 along with high expression of immune-regulatory cytokines IL-10 and TGF-β that counteract the macrophage activation with enhanced parasite survival and disease progression (23–25). Importantly, a range of cutaneous manifestations are seen in humans with differences in the number and nature of lesions, parasite load and persistence, and localized versus diffuse lesions with varying timelines of healing. The heterogeneity in CL disease phenotypes is associated with differences in lesional and systemic host immune environments. The gradation of T-cell immune response in a non-healing to healing lesional phenotype varies from a mixed Th1/Th2 to a Th1-dominant response with the key modulation exerted by the regulatory cytokines IL-10 and TGF-β (19). The regulatory cytokine axis controls the host antiparasitic response by differential modulation of pro-inflammatory and anti-inflammatory cytokine levels that may exhibit as subclinical infection to mild-to-moderate disease to extreme CL phenotypes (19, 26). In classical CL cases, susceptibility is represented by non-healing lesions with low IFN-γ and high IL-4 as well as IL-10 production and vice versa in cases with healing lesions, highlighting the significance of IFN-γ/IL-10 ratio in driving disease phenotype and severity (17, 27–29).

With this background, the immune status of patients designated as “Atypical CL cases” caused by *L. donovani* is not yet explored substantially with only a few preliminary reports on lesional cytokine patterns in atypical CL patients from Sri Lanka (30–32). These reports imply a Th1-biased lesional cytokine environment with co-expression of Th2 cytokines such that the levels of the IFN-γ correlate with the healing versus non-healing lesion duration. In this regard, systemic immune correlates of the atypical CL patients are not yet understood. Thus, the manifestation of *L. donovani*-mediated atypical CL (LdCL) needs to be urgently explored from an immunological perspective with the potential to explain the unusual disease phenotype. In the present work, we investigated systemic immune-cytokine expression profile and parasite-specific immunoglobulin G (IgG) and IgG subclasses expression patterns in LdCL patients from the endemic state of HP (11). The study provides new knowledge on host immune variables as determinants of restricting a classically visceralizing *L. donovani* to localized cutaneous lesions unlike the systemic infection in northeast India. In turn, the study brings out the significance of the fine-tuning of the host immune response modulated by the *L. donovani* variants circulating in the study region in driving the cutaneous outcome.

## Materials and Methods

### Study Samples and Ethics Statement

The clinically confirmed CL patients with respective endemic controls from HP and VL patients with endemic controls from Muzaffarpur, Bihar, were enrolled in the present study. Blood samples were collected from the CL patients reporting at the Department of Dermatology, Indira Gandhi Medical College, Shimla (IGMC, Shimla) and Mahatma Gandhi Medical Services Complex Khaneri, Rampur (MGMSC, Rampur) at the time of diagnosis. Informed consent was obtained from all the patients and parents/guardians for minors. Patients who were not taking any medication/immune suppressants were included in the study. The protocol of the present study was reviewed and approved by the Institutional Ethics Committee IGMC, Shimla, HP, Approval no. HFW (MS) G-5 (Ethics)/2014-10886 and Central University of Punjab, Approval no. CUPB/IEC/2016/034. Blood samples from 20 CL patients (HPCL) and 18 endemic control individuals from HP (HPEC) were taken for the immune-profiling studies. Additionally, blood samples from 12 VL patients (BVL) and seven endemic healthy controls (BEC) from the northeast VL belt were collected at the Medical Research Centre, Muzaffarpur, Bihar, India, Approval no. Dean/2015-16/EC/364.

### Plasma and Serum Isolation From Blood Samples

Blood was mixed with sterile phosphate-buffered saline (PBS) buffer in a 1:1 ratio and gently layered on Ficoll (HiSep LSM 1077, LS001) with the sample-to-Ficoll ratio maintained at 2:1 ratio. After centrifugation at 400*g* for 30–40 min at room temperature (RT), the layer with peripheral blood mononuclear cells at plasma/Ficoll interface was separated, and cell-free plasma samples were stored as aliquots for ELISA-based cytokine expression analysis. For serum isolation, 1 ml of blood was collected in a 1.5-ml centrifuge tube (without anticoagulant) and processed by spinning at 3,400*g* for 10 min at RT. The serum was saved for parasite-specific total IgG and IgG subclass expression analyses.

### ELISA-Based Plasma Cytokine Expression Profiling

Plasma samples from 17 atypical CL patients (HPCL) from HP with age- and sex-matched 17 endemic controls (HPEC), 12 typical VL patients (BVL), and seven endemic controls (BEC) from Bihar were analyzed for differential expression of Th1-, Th2-, Treg-, Th17-, and Th22-specific cytokines. Cytokine expression analysis in plasma samples was performed by Sandwich ELISA as per the manufacturer’s protocol [Invitrogen: IL-6 (88-7066-22), IL-17A (88-7176-22), IL-22 (88-7522-22), TGF-β (88-8350-22), IL-1β (88-7261-22), IL-10 (88-7106-22), IFN-γ (88-7316-22), TNF-α (88-7346-22), IL-12p70 (88-7126-22), IL-4 (88-7046-22), IL-27 (88-7278-22) and IL-23 (88-7237-22)]. Briefly, ELISA plates were coated with 100 μl/well of capture antibody in coating buffer and incubated overnight at 4°C. After being washed, blocking was done with 200 μl of ELISA diluent and incubated at RT for 1 h. Test plasma samples measuring 100 μl/well and standards provided with the kit were added to the designated wells. Standards were prepared with twofold serial dilution, and samples were diluted at a 1:10 ratio using the diluent provided with the kit. A total of 100 μl of 1× ELISA diluent was included as the blank, and the plate was incubated at RT for 2 h. After being washed, the detection antibody was added to all wells and incubated at RT for 1 h. After being washed, 100 μl/well of 1× TMB solution was added and incubated at RT for 15 min followed by 100 μl/well of stop solution. Plates were read at 450 and 570 nm. Each test and control sample was tested in duplicates. The optical density (OD) values were analyzed against a standard curve obtained using four-parameter logistic (4PL) regression equation in GraphPad Prism 8.0.2 (263), for the concentration of different cytokines in plasma test versus control samples. The data sets for control versus test samples were analyzed for significance by using an unpaired t-test; p-values <0.05 were considered statistically significant.

### ELISA-Based Expression Profiling of *Leishmania*-Specific Total IgG and IgG Subclasses

Serum samples from 20 HPCL patients and 18 HPEC endemic controls along with 12 BVL patients and seven BEC endemic controls were analyzed for parasite-specific total IgG and IgG subclasses using a standard protocol with best-working titrations of soluble *Leishmania* antigen (SLA; prepared from wild-type *L. donovani* DD8 culture), serum samples, and secondary antibodies (33). A 96-well microtiter ELISA plate (Thermo Fisher Scientific, Maxisorp Nunc-Immuno Plate) was coated with 1 μg/100 μl/well of SLA, in carbonate-bicarbonate buffer, pH 9.6. Plates were incubated overnight at 4°C followed by three washes for 5 min each with PBS-T (0.05% of Tween-20, Sigma, Darmstadt, Germany). Free binding sites were blocked with 100 μl of 1% bovine serum albumin (BSA) from Sigma, Darmstadt, Germany, for 1 h at RT and were washed three times with PBS-T. Serum samples measuring 100 μl at 1:2,000 dilution in PBS-T were added in sample wells along with the inclusion of appropriate positive and negative control wells. Plates were incubated for 1 h at RT and then washed three times with PBS-T. Peroxidase-conjugated mouse anti-human IgG–horseradish peroxidase (HRP) (Thermo, 054220, Waltham, MA, USA), with 1:1,000 dilution, was added at 100 μl/well. The plates were incubated for 1 h. To estimate IgG subclass isotypes, HRP-mouse anti-human IgG subclass monoclonal antibodies, anti-IgGl (Thermo, A10648, Waltham, MA, USA), anti-IgG2 (Thermo, 050520, Waltham, MA, USA), anti-IgG3 (Thermo, 053620, Waltham, MA, USA), and anti-IgG4 (Thermo, A10654, Waltham, MA, USA) were used at 1:1,000 dilution. Plates were washed three times. Enzyme substrate, TMB (Thermo, Waltham, MA, USA), measuring 50 μl/well was added to each well, and the reaction was left to proceed in the dark for 20–30 min and stopped by addition of 100 μl/well of 2N H_2_SO_4_. The endpoint was measured at 450 and 570 nm as background in an ELISA plate reader (BioTek Synergy H1, Winooski, VT, USA). OD values for healthy versus patient samples were plotted and analyzed using GraphPad Prism 8.0.2 (263). The data sets were tested for significance by using an unpaired t-test; p-values <0.05 were considered statistically significant.

## Results

### Study Groups

All CL patients enrolled in the study were indigenous to HP with no travel history to places endemic for leishmaniasis. The patients represented clinically confirmed cases exhibiting LD bodies along with histopathological changes specific to CL lesions and/or positive for *L. donovani* as the causative parasite based on species-specific PCR analysis using the lesion biopsy (11). CL and VL patients and the endemic control participants included in the study were grouped on the basis of age, sex, lesion duration, and rK39 serotyping (**Table 1**). An exploration of the systemic cytokine effector response comprising Th1 signature cytokines IFN-γ, TNF-α, and IL-12; Th2-specific cytokine IL-4; regulatory cytokines IL-10, IL-27, and TGF-β; and Th17 and Th22 cytokines comprising IL-6, IL-23, IL-1β, IL-17A, IL-22, and TNF-α along with parasite-specific total IgG and IgG subclasses was done for the specified study groups to gauge the immunological basis of the atypical CL caused by *L. donovani* genetic variant circulating in the study region.

**Table 1:** Baseline characteristics of Cutaneous Leishmaniasis and Visceral Leishmaniasis patients.

Age groups	HPEC (n = 18)	HPCL (n = 20)	CL lesion duration	*rk39 sero positivity of CL patients	
**Male**				**+ve**	**-ve**
0-20 yrs	3	4	2 months	3	1
21–40 yrs	3	4	4 months – 2.6 yrs	3	1
41–60 yrs	3	3	2–4 months	0	3
**Female**					
0-20 yrs	3	3	3–5 months	1	2
21–40 yrs	3	3	1–6 months	2	1
41–60 yrs	3	3	2 months–1.6 yrs	1	2
	BEC (n = 7)	BVL (n = 12)		*rk39 sero positivity of VL patients	
**Male**				**+ve**	**-ve**
0-20 yrs	1	3	–	3	0
21–40 yrs	3	2	–	2	0
41–60 yrs		2	–	2	0
**Female**					
0-20 yrs		1	–	1	0
21–40 yrs	3	3	–	3	0
41–60 yrs		1	–	1	0

HPEC, healthy controls from Himachal Pradesh; HPCL, atypical cutaneous leishmaniasis patients from Himachal Pradesh; BEC, healthy controls from Bihar; BVL, visceral leishmaniasis patients from Bihar.

*Anti-rK39 antibody detection in the sera of cutaneous leishmaniasis (CL) and visceral leishmaniasis (VL) patients.

### Th1 Dominating Cytokine Response Denominates Atypical Cutaneous Leishmaniasis

**Figure 1:**
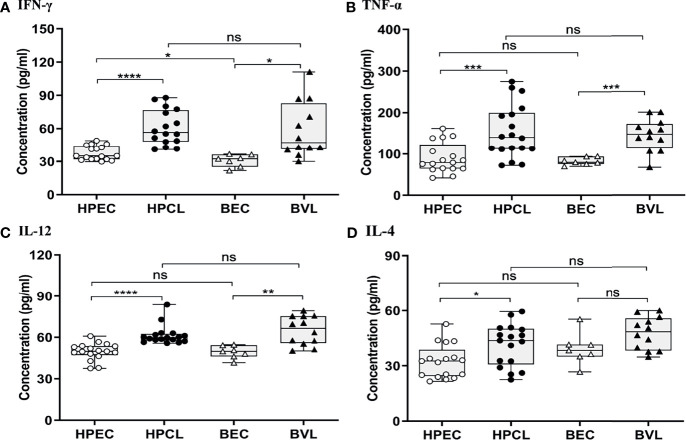
Th1/Th2-specific cytokine expression profiling of HPCL patients in comparison with BVL patients with respective endemic healthy controls. **(A)** IFN-γ; **(B)** TNFα; **(C)** IL-12; and **(D)** IL-4. Statistically significant differences are indicated by *p < 0.05, **p < 0.01, ***p < 0.001, and ****p < 0.0001; ns, non-significant; HPEC, healthy controls from Himachal Pradesh; HPCL, atypical cutaneous leishmaniasis patients from Himachal Pradesh; BEC, healthy controls from Bihar; BVL, visceral leishmaniasis patients from Bihar.

The atypical CL patients with *L. donovani* infection (HPCL) showed a significant upregulation of all the Th1 cytokines IFN-γ (p < 0.0001), TNF-α (p < 0.001) and IL-12 (p < 0.0001) compared with regional healthy control group (HPEC) (**Figures 1A–C** and **Table 2**). Similar to that in CL patients, expression of IFN-γ (p < 0.01), TNF-α (p < 0.001), and IL-12 (p < 0.01) was also found to be significantly increased in VL patients in comparison with the regional healthy controls from Bihar (**Figures 1A–C** and **Table 2**). IL-4 cytokine was found to be significantly upregulated in HPCL patients compared with the regional healthy controls (p < 0.05), while a trend towards an increase albeit non-significant was observed in VL patients in comparison with the respective endemic control group (p = 0.057) (**Figure 1D** and **Table 2**). Importantly, the Th1–Th2 cytokines analyzed showed no significant change in HPCL patients compared with BVL patients. Control samples from the two regions exhibited comparable expression of all the cytokines except IFN-γ with a trend towards higher expression in HP versus Bihar control samples (p < 0.05). Thus, *L. donovani* CL and VL cases from the two regions exhibited a predominating Th1 type of immune response with a low Th2-specific IL-4 expression. With the given cytokine readouts in the atypical CL and typical VL patients, the disease outcome cannot be assessed based on the conventional Th1–Th2 paradigm alone (14, 16, 17, 21, 22). Additional cytokines known to impinge on the Th1–Th2 axis that modulate parasite survival and dissemination were studied to understand the differential disease outcome.

**Table 2:** Cytokine expression profile of CL patients from Himachal Pradesh and VL patients from Bihar in comparison with endemic controls.

Cytokines	HPEC (n = 17)	HPCL (n = 17)	p-Value	BEC (n = 7)	BVL (n = 12)	p-Value
**Th1/Th2-specific cytokines (pg/ml)**						
**IFN-γ**	37.57 ± 1.5	67.56 ± 7.6	<0.0001 (****)	30.8 ± 2.1	58.7 ± 7.2	0.01 (*)
**TNF-α**	104 ± 16.3	165.7 ± 18	0.001 (***)	82.1 ± 3.4	145 ± 11.2	0.0006 (***)
**IL-12**	49.72 ± 1.5	61.51 ± 1.7	<0.0001 (****)	49.3 ± 1.7	65.05 ± 3	0.0015 (**)
**IL-4**	32.34 ± 2.2	41.38 ± 2.7	0.015 (*)	39.2 ± 3.2	47.8 ± 2.6	0.057 (ns)
**Th17- and Th22-specific cytokines (pg/ml)**						
**IL-17A**	35.50 ± 1.5	40.62 ± 2.5	0.094 (ns)	40.19 ± 2	49 ± 3.2	0.07 (ns)
**IL-22**	189 ± 11.8	291.9 ± 23	0.0004 (***)	207 ± 33.8	491.4 ± 52	0.0013 (**)
**IL-6**	19.84 ± 2.6	34.87 ± 3.1	0.024 (*)	21.2 ± 3.9	26.6 ± 5.3	0.47 (ns)
**TGF-β**	155 ± 26.5	180 ± 35.3	0.96 (ns)	189 ± 29.6	298.1 ± 38	0.064 (ns)
**IL-1β**	46.28 ± 2.2	68.18 ± 6.3	0.002 (**)	48.8 ± 7.2	67.5 ± 7.5	0.12 (ns)
**IL-23**	56.49 ± 1.4	58.16 ± 1.5	0.42 (ns)	51.8 ± 3.2	69.6 ± 2.9	0.0011 (**)
**Regulatory cytokines and IFN-γ/IL-10 ratio (pg/ml)**						
**IL-10**	51.65 ± 2.4	44.15 ± 2.2	0.03 (*)	50.5 ± 9.4	142 ± 9.8	<0.0001 (****)
**IL-27**	244.6 ± 8.6	278.7 ± 9.7	0.013 (*)	260.9 ± 16	274 ± 18	0.633 (ns)
**IFN-γ/IL-10 ratio**	0.724 ± 0.03	1.48 ± 0.12	<0.0001 (****)	0.733 ± 0.13	0.427 ± 0.05	0.019 (*)

HPEC, healthy controls from Himachal Pradesh; HPCL, atypical cutaneous leishmaniasis patients from Himachal Pradesh; BEC, healthy controls from Bihar; BVL, visceral leishmaniasis patients from Bihar; CL, cutaneous leishmaniasis; VL, visceral leishmaniasis.

Statistical significance is indicated by *p < 0.05, **p < 0.01, ***p < 0.001, and ****p < 0.0001; ns, non-significant.

### Th17/Th22-Mediated Effector Functions Correlate With Atypical Localized Cutaneous Leishmaniasis

**Figure 2:**
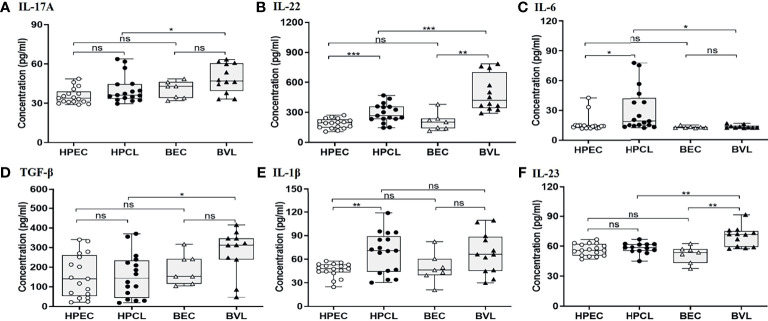
Th17/Th22-specific cytokine expression profiling of HPCL patients in comparison with BVL patients with respective endemic healthy controls. **(A)** IL-17A; **(B)** IL-22; **(C)** IL-6; **(D)** TGF-β; **(E)** IL-1β; and **(F)** IL-23. Statistically significant differences are indicated by *p < 0.05, **p < 0.01, ***p < 0.001, and ****p < 0.0001; ns, non-significant; HPEC, healthy controls from Himachal Pradesh; HPCL, atypical cutaneous leishmaniasis patients from Himachal Pradesh; BEC, healthy controls from Bihar; BVL, visceral leishmaniasis patients from Bihar.

Atypical CL patient group (HPCL) exhibited a marginal Th17 response with no significant increase in IL-17A (p = 0.094), IL-23 (p = 0.42), and TGF-β (0.96) albeit with a significant increase in the levels of IL-22 (p < 0.001), IL-1β (p < 0.01), and IL-6 (p < 0.05) compared with the HPEC control group (**Figures 2A–F**, **Table 2**). Importantly, a significant increase in expression of TNF-α, IL-6, and IL-22 implied an enhanced Th22-mediated antiparasitic and tissue-protective response in the atypical CL cases (**Figures 1B**, **2A–C**). In VL patients, a trend towards an increase in IL-17A (p = 0.07), TGF-β (p = 0.064), and IL-1β (p = 0.12) levels, albeit non-significant, was seen along with a low IL-6 level (p = 0.47) comparable with the endemic control HPEC group. However, a significant elevation in IL-22 (p < 0.01) and IL-23 (p < 0.01) expression patterns was observed in the same group (**Figure 2** and **Table 2**). The control samples from the two regions showed no significant difference in expression of all the Th17/Th22 cytokines analyzed. However, the HPCL samples exhibited significantly decreased levels of IL-17A (p < 0.05), IL-22 (p < 0.001), IL-23 (p < 0.01), and TGF-β (p < 0.05) with no significant change in IL-1β and a moderately significant increase in IL-6 levels (p < 0.05) compared with BVL patient group. Of interest, the Th17/Th22 cytokine pattern in both the CL and VL samples was associated with a significant increase in the antiparasitic IFN-γ expression (**Figure 1A** and **Table 2**). An assessment of the key regulatory cytokines was further performed as the spectrum of Th17/Th22 cytokines work together in a regulatable circuitry with pleiotropic immune functionality.

### Downregulation of IL-10 With a High IFN-γ/IL-10 Ratio Underlines the Atypical Cutaneous Leishmaniasis

**Figure 3:**
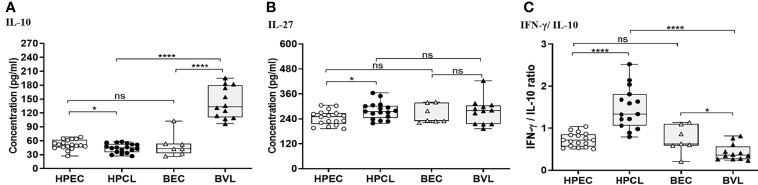
T regulatory cytokine expression profiling of HPCL patients in comparison with BVL patients with respective endemic healthy controls. **(A)** IL-10. **(B)** IL-27. **(C)** IFN-γ/IL-10 ratio. Statistically significant differences are indicated by *p < 0.05, **p < 0.01, ***p < 0.001, and ****p < 0.0001; ns, non-significant; HPEC, healthy controls from Himachal Pradesh; HPCL, atypical cutaneous leishmaniasis patients from Himachal Pradesh; BEC, healthy controls from Bihar; BVL, visceral leishmaniasis patients from Bihar.

Cytokines with immune-regulatory functions, viz., IL-10, IL-27, and TGF-β, modulate antiparasitic immunity and host tissue pathology and thus control the disease outcome. IL-10 expression analysis in the atypical HPCL patients exhibited a significant decrease (p < 0.05) in comparison with the HPEC control group, while the expression was significantly upregulated in VL patients (p < 0.0001) in comparison with the respective regional control group (**Figure 3A** and **Table 2**). Interestingly, the CL cases from HP showed a significant decrease in plasma levels of IL-10 compared with VL cases from Bihar (p-value <0.0001) with no change in the expression levels of the cytokine in the control samples from the two regions. The decreased IL-10 production along with an enhanced IFN-γ expression induced by the *L. donovani* variant in HPCL patients corresponded with a significantly high IFN-γ/IL-10 ratio (p < 0.0001) compared with BVL cases (p < 0.05) that could explain the residual systemic circulation of the parasite with a cutaneous manifestation over a systemic VL infection (**Figure 3C** and **Table 2**). TGF-β showed no significant change in the HPCL group (p = 0.96) as well as the BVL group (p = 0.064) compared with the respective control groups albeit with a significant decrease (p < 0.05) in the atypical CL cohort compared with the typical VL patients. IL-27, a pleiotropic cytokine with a discrete regulatory role in IFN-γ-, IL-17-, and IL-10-mediated immune processes exhibited a trend towards a significant increase in the HPCL group (p < 0.05) compared with the HPEC control group while BVL patients showed a comparable IL-27 expression (0.633) with respect to the BEC group (**Figure 3B** and **Table 2**).

### A Strong Humoral Response With Enhanced IgG1, IgG2, and IgG3 in Atypical Cutaneous Leishmaniasis

The systemic presence of the parasite in the HPCL patients based on anti-rK39 seropositivity and/or ITS1 PCR-based detection suggested a robust humoral response elicited by the dermotropic *L. donovani* variant (11). We assessed ELISA-based levels of anti-*Leishmania*-specific total IgG and IgG subclasses in HPCL and BVL patients.

**Figure 4:**
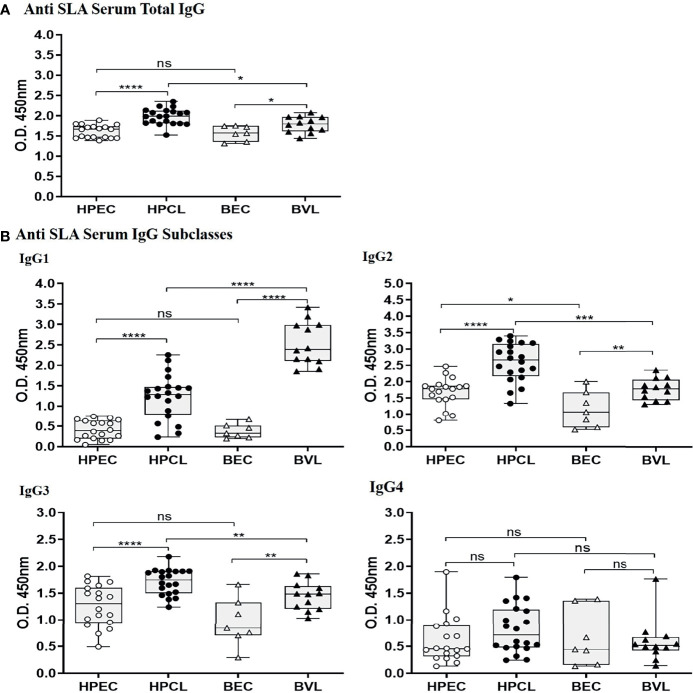
*Leishmania* antigen-specific IgG and IgG subclasses in sera from HPCL and BVL patients in comparison with respective endemic controls. **(A)** Anti-SLA-specific serum total IgG; **(B)** anti-SLA-specific serum IgG subclasses, viz., IgG1, IgG2, IgG3, and IgG4. Statistical significance is indicated by *p < 0.05, **p < 0.01, ***p < 0.001, and ****p < 0.0001; ns, non-significant; HPEC, healthy controls from Himachal Pradesh; HPCL, atypical cutaneous leishmaniasis patients from Himachal Pradesh; BEC, healthy controls from Bihar; BVL, visceral leishmaniasis patients from Bihar.

In HPCL patients, a significant increase in the levels of anti-SLA total IgG (p < 0.0001) was observed in comparison with the HPEC control group. In terms of IgG subclasses, significant elevation in levels of IgG1, IgG2, and IgG3 (p < 0.0001 for all three) with comparable IgG4 level (p = 0.157) was obtained in HPCL patients compared with the HPEC control group. Similarly, anti-SLA-specific total IgG (p < 0.05) and IgG subclasses, viz., IgG1 (p < 0.0001), IgG2 (p < 0.01), and IgG3 (p < 0.01) levels, were significantly higher in BVL patients compared with the BEC control group with comparable IgG4 level (p = 0.795) in line with an earlier report by Ghosh et al. (**Figures 4A, B** and **Table 3**) (34). Overall, parasite-specific total IgG level was significantly higher in HPCL patients compared with BVL patients with the increase majorly reflected in IgG2 and IgG3 levels along with a decrease in IgG1 levels and no significant change in IgG4 levels (**Figures 4A, B** and **Table 3**). The finding on differences in the SLA-specific IgG and IgG subclasses between the HPCL and BVL patients represents the possible differences in the immunogenic antigens of the atypical CL-causing and VL-causing *L. donovani* strains. The data clearly represent a robust antibody response in atypical CL patients with the previously demonstrated circulatory load of the parasite (11).

**Table 3:** Comparison of *Leishmania* antigen-specific total serum IgG and IgG subclasses in CL and VL patients along with respective endemic controls.

IgG	Total IgG (OD)	p-Value	IgG1 (OD)	p-Value	IgG2	p-Value	IgG3 (OD)	p-Value	IgG4 (OD)	p-Value
**HPEC**	1.62 ± 0.04 n = 18	(<0.0001)****	0.42 ± 0.05 n = 18	(<0.0001)****	1.68 ± 0.11 n = 18	(<0.0001)****	1.26 ± 0.09 n = 18	(<0.0001)****	0.61 ± 0.11 n = 18	ns (0.157)
**HPCL**	1.98 ± 0.05 n = 20		1.23 ± 0.13 n = 20		2.59 ± 0.13 n = 20		1.72 ± 0.05 n = 20		0.83 ± 0.1 n = 20	
**BEC**	1.57 ± 0.07 n = 7	(0.0335)*	0.38 ± 0.07 n = 7	(<0.0001)****	1.15 ± 0.21 n = 7	(0.007)**	0.95 ± 0.17 n = 7	(0.0077)**	0.65 ± 0.19 n = 7	ns (0.795)
**BVL**	1.78 ± 0.06 n = 12		2.53 ± 0.15 n = 12		1.77 ± 0.09 n = 12		1.45 ± 0.07 n = 12		0.59 ± 0.12 n = 12	

HPEC, healthy controls from Himachal Pradesh; HPCL, atypical cutaneous leishmaniasis patients from Himachal Pradesh; BEC, healthy controls from Bihar; BVL, visceral leishmaniasis patients from Bihar; CL, cutaneous leishmaniasis; VL, visceral leishmaniasis.

Statistical significance is indicated by *p < 0.05, **p < 0.01, ***p < 0.001, and ****p < 0.0001; ns, non-significant.

## Discussion

Leishmaniasis initiates as a skin infection and progresses as varied disease outcomes, driven by the infecting *Leishmania* species/strains and heterogeneous host immune modality. The initial host immune response elicited by distinct parasite determinants set the stage for differential parasite persistence, dissemination, and immunopathogenesis that together determine the gradation of visceral and cutaneous manifestations. Further, with *Leishmania* being an intracellular pathogen, the total of T-cell immunity comprising heterogeneous T-cell subsets with discrete effector cytokines direct the course of the disease ranging from non-symptomatic to mild-to-severe VL and CL phenotypes (14, 16, 17, 21, 22). With substantial insight into the host immune correlates that underline the classical visceral and cutaneous manifestation, scarce reports are available on host immune correlates in *L. donovani* atypical CL disease emerging in known and newer endemic sites in the Indian subcontinent (10, 30–32, 35, 36). Knowledge on comparative host immune response in LdCL versus typical VL is important to define the key immune checkpoints that can modulate the disease towards atypical phenotype (9–12, 37). In this regard, no data are available on the systemic cytokine correlates in atypical CL cases with few reports on RT PCR-based lesional cytokine environment in Sri Lankan atypical CL cases. Our finding on systemic cytokine expression in atypical HPCL patients is in line with a Th1-dominating lesional cytokine response reported earlier in Sri Lankan CL patients with elevated levels of plasma IFN-γ, TNF-α, and IL-12 along with a low-to-undetectable expression of IL-4 (30–32). With the knowledge that IFN-γ is the key Th1 cytokine critical in the defense against the intracellular parasite, a robust Th1 response in HPCL patients suggests an enhanced parasite killing activity that could result in parasite restriction to localized skin lesions (17, 21). In contrast, the Th2-specific IL-4 is a disease-promoting cytokine and counteracts protective Th1 response, which further supports dominance of Th1 parasite killing systemic response in HPCL patients exhibiting a marginal increase in IL-4 (17, 21). Importantly, with the Th1 protective response similar to the typical VL cases albeit with cytokine-specific quantitative differences, the expression pattern of additional cytokines could possibly explain the dermotropic manifestation in HPCL cases.

**Figure 5:**
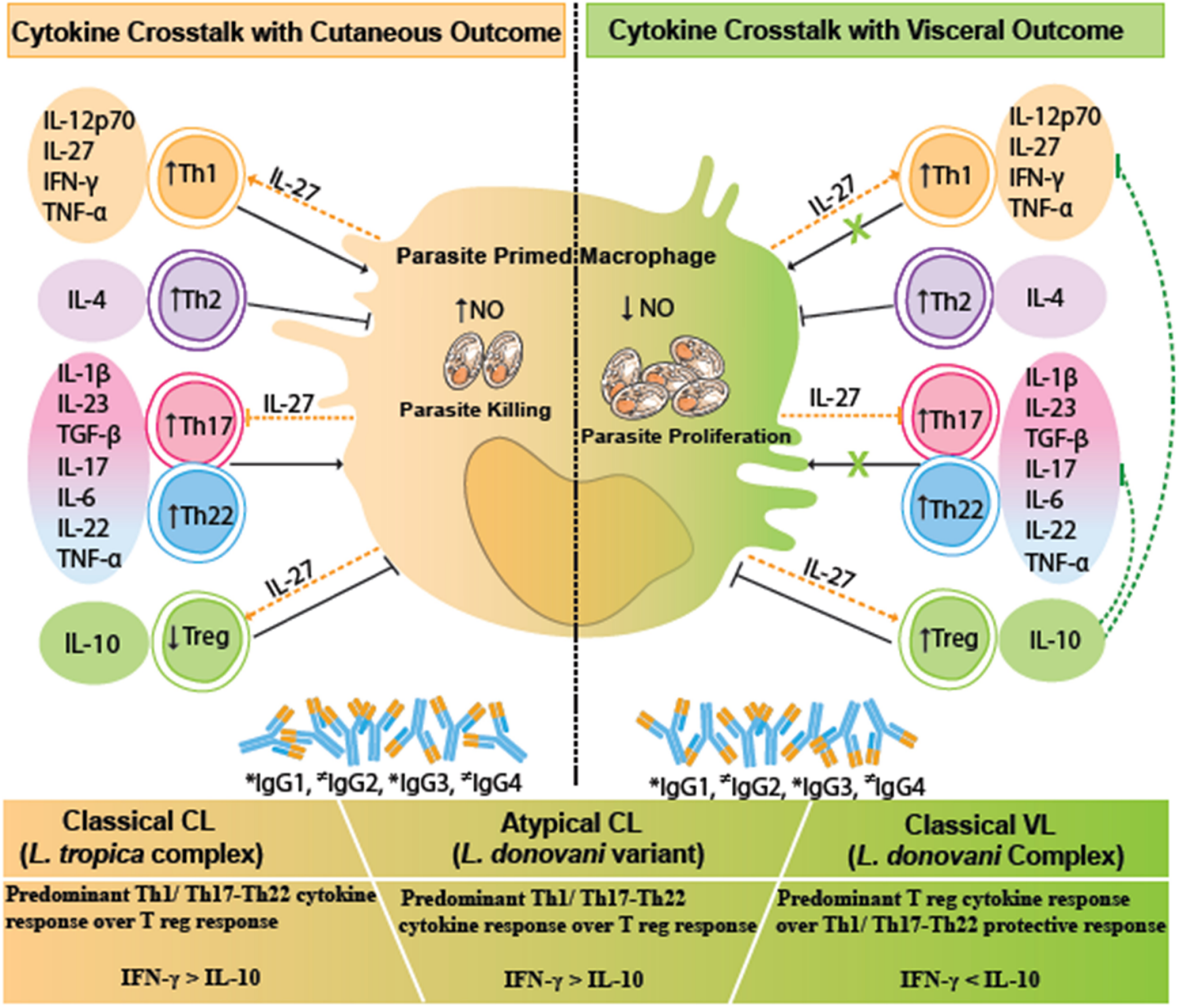
A comparative cytokine immune crosstalk in different human leishmaniasis pathologies, viz., VL, CL, and atypical CL. Upon parasitic encounter, there is a significant release of Treg cytokines especially IL-10, along with Th1/Th17/Th22 cytokines, and increased levels of IgG1, IgG2, and IgG3 antibodies in VL. IL-10 counteracts the protective Th1/Th17/Th22 cytokine response with a resultant increase in parasite proliferation and disease progression in VL. CL is marked by a significant Th1 type of immune response and Th22 tissue repair response along with the increase in IgG1, IgG2, and IgG3 antibodies. IL-27 helps in parasite killing *via* facilitating Th1 cytokine response. The given immune correlates in cutaneous disease halts the *Leishmania* parasite proliferation with a disease protective response. Immune correlates in atypical CL are skewed towards a CL type of immune response. Dotted green lines represent IL-10-mediated inhibition of antiparasitic and immunopathological effector function of Th1 and Th17/Th22-specific cytokines. Dotted orange lines represent the pleiotropic role of IL-27-mediated induction of Th1 and Treg activity along with inhibition of Th17 response. * indicates increased levels of IgG isotypes; ^≠^ indicates variable levels of IgG isotypes. VL, visceral leishmaniasis; CL, cutaneous leishmaniasis.

In this regard, Th17/Th22-specific pro-inflammatory cytokines are important regulators of parasite multiplication and ensuing tissue inflammation that determine heterogeneous outcomes in VL and CL (15, 19, 38–40). The Th17–Th22 antiparasitic cytokine axis works in a cross-regulatory loop such that Th17 effector cytokines lead to tissue inflammation if over-produced, and Th22 effector response is understood more as tissue-protective (41–43). Th17 effector function itself exhibits heterogeneity with an inflammatory Th17 pathogenic response that synergizes with parasite-killing Th1 activity or a regulatory-type Th17 response that works to maintain immune homeostasis (41, 44). Additionally, Th22 effector cytokines crosstalk with the Th17 cells *via* the common pool of IL-22 effector cytokine (**Figure 5**). IL-22 is antiparasitic with a predominant role in tissue repair along with the potential to mediate the inflammatory response in conjunction with IL-17 (17, 42, 43). Thus, differential expression of Th17 and Th22 cytokines drives the degree of microbicidal activity, host tissue inflammation, and repair in varied CL and VL manifestations. A basal Th17 and an enhanced Th22 response in atypical CL patients could thus manifest antiparasitic and tissue-protective responses albeit with limited inflammation in the presence of marginal IL-17 level. Additionally, an enhanced IL-1β itself can induce parasite-killing NO production similar to TNF-α and can suppress IL-4 activity, while IL-6 can skew the IL-22-mediated antiparasitic and tissue repair response. The said cytokine pattern can possibly explain the development of the localized cutaneous lesions associated with low parasite load and restricted tissue damage (41, 42). In CL, the Th17/IL-17 and Th22/IL-22 axes are reported to be parasite species-dependent such that varying spectra of lesional and systemic Th17/Th22 cytokine expression pattern correlate with heterogeneous phenotypes of healing to non-healing cutaneous lesions (19). This can possibly implicate the threshold of host Th17/Th22 cytokine pattern induced by an antigenic pool of *L. donovani* variant in the study region to be responsible for the atypical localized cutaneous manifestation.

HPCL patients exhibited a near-around cytokine pattern in comparison with the VL cases with respect to Th1/Th17/Th22 cytokines, although with dramatically different disease outcomes. The phenomenon can be explained on the basis of the difference in the expression levels of the regulatory cytokine IL-10. IL-10 is demonstrated to be the key immunoregulatory cytokine that pins down the protective function and regulates inflammation mediated by the Th1/Th17/Th22-specific cytokines, thereby modulating the parasite load and host immuno-pathological consequences as shown in **Figure 5**. The regulatory role of IL-10 in varied spectra of visceral and cutaneous disease manifestations is mediated by its differential induction by the infecting parasite species/strains and the host genetics (17–19, 24, 45, 46).

Classical visceral disease is associated with elevated levels of IL-10 that mediate systemic parasite dissemination with varying degrees of tissue damage correlating with disease severity (23, 47–52). The higher IL-10 expression in the BVL patient group, in line with reports on classical VL, could render the Th1/Th17/Th22 cytokine axis ineffective in parasite killing and could lead to visceral dissemination and aggravated host tissue damage (24, 25, 41, 48, 53, 54). In the cutaneous disease, a varied expression of IL-10 lesser than the levels in typical VL disease is reported, with a role in limiting parasite load as well as persistence along with the extent of tissue damage in varying gradations of lesion types and severity (17, 19, 26, 46, 55, 56). With this understanding, a significant downregulation of IL-10 in HPCL patients compared with the VL group suggests an effective parasite-killing Th1 axis that could limit the systemic parasite circulation supported by the detection of faint *L. donovani*-specific amplicons with lack of any VL like diagnosis (11). The central role of IL-10 proposed in modulating VL to CL outcome in the HPCL patients is strengthened by the reports on typical VL and CL diseases, wherein the phenotypic heterogeneity from systemic to cutaneous disease with healing to non-healing lesions correlates with the ratio of protective IFN-γ to regulatory IL-10 levels (19, 24) (**Figure 5**). Also, suppressed TGF-β expression in the atypical CL cohort compared with the typical VL patients signifies an enhanced parasite killing activity of host cells.

IL-27 regulates IFN-γ-mediated Th1 protection, enhances IL-10-mediated regulatory response, and also functions as a negative regulator of Th17 inflammatory response (54, 57–64). A trend towards an increased IL-27 expression in HPCL could imply its role in an altered Th1–Treg–Th17 axis such that localized cutaneous lesions with limited cutaneous pathology and parasite load are manifested in corroboration with the few CL-specific reports available (60, 65). IL-27 expression in the BVL patients was comparable with that in the control group in contrast to the existing reports for elevated IL-27 in VL patients, possibly due to the small sample size with variable reads (54, 66).

Assessment of parasite-specific humoral response in terms of the elevated IgG and differential titers of *Leishmania*-specific IgG subclasses is another important immune correlate of the disease phenotype. With a lack of understanding of the protective role of antibodies, augmented IgG antibody response against a parasite is reported for both VL and CL with variation in IgG subclasses driven by cytokine-mediated isotype class/subclass switching (67–70). In typical VL and CL cases, SLA-specific IgG along with IgG1 and IgG3 have been shown to be enhanced with variable reports on IgG2 and IgG4 expression patterns (34, 71–74). With the lack of such study for the atypical CL cases, we elucidated a robust parasite-specific IgG response in CL patients from HP with values higher than the typical VL patients. Interestingly, the relative increase in total IgG levels in HPCL versus BVL cases majorly represented enhanced IgG2 and IgG3 levels with decreased IgG1 levels. The pattern of the differential IgG1, IgG2, and IgG3 levels in HPCL versus BVL patients possibly represents differences in the complement of B cell-specific immunodominant antigens in the CL- and VL-causing *L. donovani* variants (68, 70). This is reflected in the poor rK39 seroreactivity of atypical CL patients in the study area along with similar findings with Sri Lankan CL cases (11, 33). It is important to mention that IgG/IgG subclass evaluation in the present study was done using SLA prepared using a standard VL-derived *L. donovani* culture. An enhanced antibody titer suggests the presence of a large number of common immuno-dominant antigens in the VL-causing *L. donovani* variant and CL-causing *L. donovani* variant along with antigenic variations showing up in terms of differences in levels of IgG subclasses and rK39 reactivity (11). With IL-10 determining the IgG1 and IgG3 isotype switching, variation in levels of IL-10 in HPCL and BVL patients could potentially result in differences in the levels of IgG subclasses with a role in downstream effector functions potentially associated with different disease outcomes.

## Conclusion

In summary, an enhanced IFN-γ/IL-10 ratio in atypical CL cases could potentially explain the localized cutaneous phenotype over the visceral disease. IL-27 could potentially cross-regulate the Th1/Th17/Th22-mediated spectrum of immune effector function associated with heterogeneous disease outcomes as depicted in **Figure 5**. Importantly, we can conclude that IL-10 is the key immune checkpoint that potentially modulates the disease phenotype towards a cutaneous manifestation. With a decrease in IL-10, an effective Th1/Th17/Th22 response would ensue that could restrict the *L. donovani* variant to the skin and modulate the disease progression towards a cutaneous phenotype in the HPCL cases over a visceral phenotype that is classically associated with high circulatory IL-10 levels. An altered pool of immunogens, evident from the multiple genetic changes within the coding genomic sequences in the atypical *L. donovani* isolates in comparison with the classical wild-type *L. donovani*, could possibly fine-tune the systematic immune response towards a cutaneous phenotype. Importantly, for an understanding of the mechanism of IL-10-mediated atypical cutaneous phenotype, the cause–effect axis needs to be explored further. In-depth analysis of the immune cells with discrete cytokine expression profiles, possibly mediating the observed cytokine changes, needs to be studied by deep flow cytometry profiling. Advances in the understanding of T-cell immunity in terms of the T-cell subsets that produce specific cytokines along with the target cells with cognate cytokine receptors can give major leads towards elucidating atypical cases from an immunological perspective. Such findings will be relevant with the preposition for rationalized immune therapeutics and vaccine development against the atypical disease *per se* along with the visceral and cutaneous diseases. Thus, more studies for unraveling the mechanism of the key cytokine players involved in atypical CL manifestation are required. An animal model mimicking atypical CL can provide a workable model to further dissect out the immune checkpoints that drive the unusual disease phenotype along with a better definition of the complex immune environment that drives VL and CL.

## Data Availability Statement

The original contributions presented in the study are included in the article/supplementary material. Further inquiries can be directed to the corresponding author.

## Ethics Statement

The studies involving human participants were reviewed and approved by Indira Gandhi Medical College, Approval no. HFW (MS) G-5 (Ethics)/2014-10886; Central University of Punjab, Approval no. CUPB/IEC/2016/034; and Medical Research Centre, Muzaffarpur, Bihar, India Approval no. Dean/2015-16/EC/364. Written informed consent to participate in this study was provided by the participants’ legal guardian/next of kin.

## Author Contributions

LT collected the samples, performed the experiments, and analyzed the data. PM helped with the figures. VS and AN provided the CL samples. AJ provided infrastructural support to execute the experiments. SBC, OP, and SS provided the VL samples. OP and AJ critically revised the manuscript. MJ contributed to the concept and design of the study, data analysis, drafting of the article, and final approval of the manuscript. All authors contributed to the article and approved the submitted version.

## Funding

This work was supported by Collaborative Research Project Grant from ICGEB, Trieste (Project CRP/IND19-01, GP#163, Central University of Punjab) to MJ. LT was funded with a doctoral fellowship, Central University of Punjab, Bathinda. PM is supported by DBT Senior Research Fellowship.

## Conflict of Interest

The authors declare that the research was conducted in the absence of any commercial or financial relationships that could be construed as a potential conflict of interest.

## Publisher’s Note

All claims expressed in this article are solely those of the authors and do not necessarily represent those of their affiliated organizations, or those of the publisher, the editors and the reviewers. Any product that may be evaluated in this article, or claim that may be made by its manufacturer, is not guaranteed or endorsed by the publisher.
